# Two in one: cryptic species discovered in biological control agent populations using molecular data and crossbreeding experiments

**DOI:** 10.1002/ece3.2297

**Published:** 2016-07-30

**Authors:** Iain D. Paterson, Rosie Mangan, Douglas A. Downie, Julie A. Coetzee, Martin P. Hill, Ashley M. Burke, Paul O. Downey, Thomas J. Henry, Stephe G. Compton

**Affiliations:** ^1^ Department of Zoology and EntomologyRhodes UniversityPO Box 94Grahamstown6140South Africa; ^2^ Institute for Applied EcologyUniversity of CanberraCanberraAustralian Capital Territory2601Australia; ^3^ Systematic Entomology LaboratoryARS, USDA, c/o National Museum of Natural HistorySmithsonian InstitutionWashingtonDistrict of Columbia20013

**Keywords:** Biological species concept, C01, DNA‐barcoding, *Eccritotarsus catarinensis*, *Eichhornia crassipes*, Inter Simple Sequence Repeats, reproductive incompatibility

## Abstract

There are many examples of cryptic species that have been identified through DNA‐barcoding or other genetic techniques. There are, however, very few confirmations of cryptic species being reproductively isolated. This study presents one of the few cases of cryptic species that has been confirmed to be reproductively isolated and therefore true species according to the biological species concept. The cryptic species are of special interest because they were discovered within biological control agent populations. Two geographically isolated populations of *Eccritotarsus catarinensis* (Carvalho) [Hemiptera: Miridae], a biological control agent for the invasive aquatic macrophyte, water hyacinth, *Eichhornia crassipes* (Mart.) Solms [Pontederiaceae], in South Africa, were sampled from the native range of the species in South America. Morphological characteristics indicated that both populations were the same species according to the current taxonomy, but subsequent DNA analysis and breeding experiments revealed that the two populations are reproductively isolated. Crossbreeding experiments resulted in very few hybrid offspring when individuals were forced to interbreed with individuals of the other population, and no hybrid offspring were recorded when a choice of mate from either population was offered. The data indicate that the two populations are cryptic species that are reproductively incompatible. Subtle but reliable diagnostic characteristics were then identified to distinguish between the two species which would have been considered intraspecific variation without the data from the genetics and interbreeding experiments. These findings suggest that all consignments of biological control agents from allopatric populations should be screened for cryptic species using genetic techniques and that the importation of multiple consignments of the same species for biological control should be conducted with caution.

## Introduction

The biological species concept defines species as interbreeding populations that are reproductively isolated from other populations (Mayr 1942, 1963; Noor 2002). Although the biological species concept is generally accepted by the majority of evolutionary biologists (Noor 2002; Coyne and Orr 2004), morphological characteristics, rather than the ability of biological entities to interbreed, are usually used to describe species (Schlick‐Steiner et al. 2007; Cook et al. 2010). The result of this incongruence between how species are defined and how new species are described is that many described species may comprise multiple biological species that appear to be morphologically indistinguishable or identical. Two or more species that have been classified as a single species due to their morphological characteristics are referred to as cryptic species (Darlington 1940).

There is growing evidence that cryptic species are much more common than was previously thought (Hebert et al. 2004a; Bickford et al. 2007). The growth in the number of recorded cryptic species is primarily due to the use of DNA‐barcoding which can successfully distinguish between what appear to be morphologically identical species based on the sequence divergence within and between species (Hebert et al. 2003). While the occurrence of many potential cryptic species have been identified using DNA‐barcoding (e.g., Wilcox et al. 1997; Gómez et al. 2002; Hebert et al. 2004a,b; Miura et al. 2005; Ward et al. 2008; Saitoh et al. 2015), few have been confirmed to be reproductively incompatible using crossbreeding experiments in combination with molecular data. For some organisms, it is not possible to conduct crossbreeding experiments, so multiple data sets combining morphological, ecological, and physiological data have been combined with genetic data to provide a better understanding of whether true biological species are present (e.g., Ross and Shoemaker 2005; Rissler and Apodaca 2007; Abad‐Franch et al. 2013; Davis et al. 2016; Dejaco et al. In press). Although these approaches are valuable, interbreeding experiments are a more direct and conclusive method of testing for reproductive incompatibility. There are very few examples of cryptic species being verified in this way. Some examples include the sweet potato whitefly, *Bemisia tabaci* (Gannadius) [Hemiptera: Aleyrodidae] (Xu et al. 2010; Liu et al. 2012), the dipteran vector of the disease leishmaniasis, *Lutzomyia longipalpis* Lutz and Neiva [Dipetra: Psychodidae] (Souza et al. 2008), and the biological control agent for cereal stem borers, *Cotesia sesamiae* (Cameron) [Hymenoptera: Braconidae] (Kaiser et al. 2015). This paper presents the first example of cryptic species confirmed as reproductively incompatible taxa nested within weed biological control agent populations.

*Eccritotarsus catarinensis* (Carvalho) [Hemiptera: Miridae] is a natural enemy of water hyacinth (*Eichhornia crassipes* (Mart.) Solms. [Pontederiaceae]) used for biological control in South Africa (Coetzee et al. 2011). The agent was collected in its native range of South America from two geographically isolated populations that are separated by a distance of over 3500 km (Taylor et al. 2011). The first collection of the agent for biological control was made in Florianopolis (Santa Catarina), Brazil in 1994, and this collection was subjected to host specificity testing and then released throughout South Africa (Hill et al. 1999). Prior to release, the Brazilian collection had been subjected to a severe genetic bottleneck event after importation into quarantine so a subsequent collection was made on the Yarapa River near Iquitos, Peru in 1999 (Cordo 1999), with the intention of interbreeding the populations for the purpose of increasing genetic diversity (Taylor et al. 2011). There is strong evidence that the negative consequences of a genetic bottleneck can be overcome by combining individuals from different source populations (Colautti et al. 2005; Lachmuth et al. 2010) so it was expected that when the two populations were allowed to interbreed, the resulting population would be a more effective agent (Taylor et al. 2011).

The Brazilian population, which was first released in South Africa in 1996, has established widely throughout the country and contributes toward the control of water hyacinth, especially during periodic outbreaks of the agent that result in the collapse of water hyacinth mats at some sites (Winston et al. 2014). Although biological control has successfully reduced the water hyacinth problem in South Africa, complete control has not been achieved (Coetzee et al. 2011). Cold temperatures (Coetzee et al. 2007a), as well as high nutrients in South African aquatic ecosystems (Coetzee et al. 2007b), are possible limiting factors to the efficacy of *E. catarinensis*. The low genetic diversity of the Brazilian population has been regarded as another factor that could be limiting success, so the more genetically diverse Peruvian population was released in 2007 with the intention of increasing the level of control provided by the agent (Taylor et al. 2011). Subsequent releases have been conducted but establishment of the Peruvian population is yet to be confirmed (Winston et al. 2014). The interactions between the two populations, and how each population performs at different temperatures, is likely to play an important role in the biological control programme against water hyacinth in South Africa and elsewhere in the world where *E. catarinensis* may be released in future (Taylor et al. 2011).

A comparison of the mitochondrial cytochrome oxidase subunit 1 gene (COI) of the Peruvian and Brazilian populations imported to South Africa indicated a surprisingly large sequence divergence of 5.2% between the populations, suggesting the possibility that they should be considered cryptic species (Taylor et al. 2011). Inter Simple Sequence Repeat (ISSR) data supported this conclusion as 29 fixed differences were observed (Taylor et al. 2011). Sequence divergences at the COI region have been investigated in 16 other species of Miridae and only one species had a mean divergence value higher than 5.2%, whereas four of the species had a maximum intraspecific divergence distance greater than this value (Park et al. 2011). These data indicate that most Miridae have lower intraspecific sequence divergence than that found between the Peruvian and Brazilian populations of *E. catarinensis* but also that there are some exceptions. Therefore, the presence of cryptic species in *E. catarinensis* cannot be solely inferred by the high sequence divergence alone.

The aim of this study was to test the hypothesis that the two populations of *E. catarinensis* are cryptic species according to the biological species concept. Morphological characters of the two populations were compared to confirm the morphological similarity of the two populations. The ability of the two populations to interbreed was then tested under no‐choice and choice conditions. Under no‐choice conditions, the insects could either attempt to reproduce with insects from the other population or not reproduce at all. Under choice conditions, the insects were given a choice of reproducing with mates from either population.

## Methods

### Morphological comparison

*Eccritotarsus catarinensis* was described from a holotype male and four male and nine female paratypes (including an allotype) collected in the state of Santa Catarina in southern Brazil (Carvalho 1948). In 2012, small series of both sexes from each *E. catarinensis* population were sent to the Systematic Entomology Laboratory ARS‐USDA, in Washington, DC, for identification by Thomas J. Henry, a specialist on the family Miridae. This was performed to confirm that both populations should be considered as *E. catarinensis* according to the current taxonomic knowledge of the group (Carvalho 1948). Based on comparison with six paratypes in the United States National Museum of Natural History, Washington, DC, the series were confirmed to be *E. catarinensis*. Subsequently, the specialist was made aware that the two series could represent cryptic species due to high levels of sequence divergence in the COI region and the large differences in the ISSR data (Taylor et al. 2011). Larger series of both populations were then sent for examination with the intention of identifying reliable morphological characteristics to separate the two populations.

Morphological parameters of specimens from the two populations were also compared with each other and with the original description of the species. The morphological parameters provided for the type specimen in the description include body length, body width, the parameters of the head (height, width, length), the length of each of the four antennal segments, and the length and width of the pronotum (Carvalho 1948). The same measurements were taken for ten males and ten females from each populations using an Olympus 52X16 compound microscope and the imaging software programme Olympus Stream Motion (Olympus Corporation Ltd, Tokyo, Japan). The clasper organs, or parameres, of male *Eccritotarsus* are important diagnostic features that are used to distinguish between the various species within the genus (Carvalho 1948). For this reason, the parameres were dissected from ten males of each population, mounted on microscope slides and examined in greater detail. The length of the left paramere and the width of the hook at the end of the left paramere were measured. Data for all morphological parameters were normally distributed so the populations were compared using *t*‐tests in STATISTICA ver. 13 (StatSoft Inc., Tulsa, OK).

### No‐choice interbreeding experiment

Late‐instar nymphs were collected from each of the populations and placed individually in Petri dishes with dampened filter paper and a water hyacinth leaf‐disk approximately 2 cm in diameter. The Petri dishes were labeled according to the population source of the nymph and sealed with Parafilm^®^ to maintain humidity. Leaf‐disks and moist filter paper were replaced when necessary. Keeping the nymphs individually until their final ecdysis to the adult stage ensured that all the individuals used in this experiment were unmated.

Within 12 h of the final ecdysis, adults were removed from the Petri dishes and divided into breeding pairs. The four breeding pair treatments were: Brazil♀ × Brazil♂ (*n* = 10); Peru♀ × Peru♂ (*n* = 15); Brazil♀ × Peru♂ (*n* = 27) and Peru♀ × Brazil♂ (*n* = 24). Each breeding pair was placed on a small water hyacinth plant in a clear plastic jar half filled with water and covered with nylon mesh. The plants were examined daily for the presence of nymphs for a 30‐day period and the total number of nymphs produced by each breeding pair was recorded.

Data were not normally distributed so a Kruskal–Wallis ANOVA and a Multiple Comparison of Mean Ranks were conducted in STATISTICA v. 13 (StatSoft Inc. 1984–2014). Hybrid offspring and the parents of hybrids were collected and preserved individually in 100% ethanol for genetic analysis to verify that they were true hybrids. Genetic analysis was conducted according to the methods of Taylor et al. (2011) using a combination of the COI mitochondrial sequence and the nuclear ISSR profile to determine whether offspring were Brazilian, Peruvian or hybrids (see Genetic analysis below for more details of genetic analysis).

### Choice interbreeding experiment

Choice tests were included because no‐choice situations are unlikely to occur under natural conditions and because no‐choice tests may produce false positive results (Liu et al. 2012).

For choice experiments, eight 70‐L tubs filled with water hyacinth and covered with a fine (0.5 mm) mesh net were inoculated with 200 *E. catarinensis* individuals. Two of the tubs were controls, one with 200 Brazilian *E. catarinensis* and the other with 200 Peruvian *E. catarinensis*. The other six tubs (referred to as replicates 1–6) were founded with 100 individuals from each population. The experiment was conducted in a greenhouse at Rhodes University, Grahamstown, South Africa, for 6 months from February to August 2011. After the 6‐month period, nine individuals from each of the tubs were randomly sampled and stored individually in 100% ethanol for genetic analysis. Adult *E. catarinensis* live for an average of about 50 days (Hill et al. 1999), so all the insects used at the start of the experiment would have been dead by the time that sampling took place. The insects sampled for genetic analysis were therefore the progeny of those used to start the experiment, not the same individuals. Extractions were performed on nine individuals from each replicate and the two controls but in some cases, extractions or PCRs failed. Sixty‐eight individuals were sampled in total, including nine from each of the two controls.

### Genetic analysis

Taylor et al. (2011) characterized the Peruvian and Brazilian populations using the COI region and ISSR profiles. Following from their results, if hybrid individuals were sampled, they would be expected to be intermediate in a nonmetric Multi‐Dimensional Scaling (nMDS) scatterplot from the nuclear (ISSR) data, and have an identical COI (mitochondrial) sequence to their mother.

A whole *E. catarinensis* adult or nymph was used for each DNA extraction. DNA was extracted, the COI region was sequenced and ISSR analysis was performed according to the methods outlined in Taylor et al. (2011). The COI region was amplified using the LCO 1490 and HCO 2198 primers (Hebert et al. 2003). ISSRs were conducted using the universal primers 809 and 826 from University of British Columbia Nucleic Acid Protein Service Unit Primer set #9 (Abbot 2001), fluorescently labeled with 6‐FAM dye. Electropherograms were analyzed using PeakScanner v. 1.0 (Applied Biosystems, Foster City, CA) using a medium level of peak smoothing. The analyzed data set was then exported into RawGeno v. 2.0‐2 (Arrigo et al. 2009) using the R© v. 3.0.1 platform (The R Foundation for Statistical Computing, Vienna, Austria). Every sample was replicated from the PCR step so there were two sets of binary data for each sample. Only peaks that were present in both replicates were used for further analyses. Bands that were only present in one of the replicates were treated as missing data. The final binary data set was then used to conduct an MDS analysis in PAST: Paleontological Statistics Package v. 1.94 (Hammer and Harper 2001) using Jaccard's Index to convert the binary data into a distance matrix.

The position of each sample on the nMDS scatterplot compiled using the ISSR data, along with the corresponding COI haplotype for that sample was used to identify whether an individual was a pure Brazilian, pure Peruvian or hybrid. The mother's population of a hybrid offspring was identified from the COI haplotype of the nymph.

### Screening for *Wolbachia*

*Wolbachia*, a bacterial endoparasite that is common in arthropods, can result in cytoplasmic incompatibility between intraspecific lineages and could therefore be a confounding factor in interbreeding experiments to confirm reproductive isolation (Bordenstein et al. 2001). For example, cytoplasmic incompatibility has been reported between infected and uninfected lineages of the predatory mirid, *Macrolophus pygmaeus* (Rambir), resulting in a reduction in the number of offspring produced when crossing infected males with uninfected females but not when uninfected males were crossed with infected females (Machtelinckx et al. 2009). *Wolbachia* has also been shown to promote speciation through bidirectional cytoplasmic incompatibility between closely related species of parasitic wasps (Bordenstein et al. 2001). If one of the *E. catarinensis* populations had a high rate of *Wolbachia* infection, or if both populations had distinct *Wolbachia* strains, then it is possible that interbreeding experiments could falsely identify the two populations as cryptic species because crosses between infected and uninfected individuals, or individuals infected with different *Wolbachia* strains, may not produce offspring. Both *E. catarinensis* populations were therefore screened for the presence of *Wolbachia*.

*Wolbachia* positive controls were sourced from an infected culture of *Eldana saccharina* Walker (Pyralidae) housed at the South African Sugar Research Association (SASRI), Mount Edgecombe, KwaZulu‐Natal, South Africa. The *wsp* 81F and 69IR primers were selected for *Wolbachia* screening. These primers amplify a 590–632 bp fragment of the *wsp* gene (Zhou et al. 1998). The *wsp* primers amplify most *Wolbachia* strains and produced visible bands for all *Wolbachia* infected arthropod species tested in a recent assessment of methods for screening for *Wolbachia* (Simões et al. 2011). Extractions were conducted as reported above for the *E. saccharina* control and twenty *E. catarinensis* from each population. Each 20 μL reaction contained 10 μL of GoTaq^®^ Colorless Master Mix, 3 μL of extracted DNA, 0.4 μL of each primer, 5.8 μL of nuclease‐free water and 0.4 μL of MgCl to give a final MgCl concentration of 2.5 mmol/L. The PCR protocol had an initial denaturing step of 94°C for 1 min followed by 32 cycles of 94°C for 1 min, 50°C for 45 sec, 72°C for 1 min and a final extension of 4 min at 72°C. The PCR products were then run for 30 min at 80 V on a 1.2% agarose gel stained with SybrSafe™ (Applied Biosystems, Foster City, CA) to visualize bands under ultraviolet light. A 100 bp DNA ladder was included to estimate the size of any fragments that were produced.

## Results

### Morphological comparison

As noted, the Brazilian and Peruvian series of specimens were all identified originally as *E. catarinensis* (Carvalho) by mirid specialist T.J. Henry (Systematic Entomology Laboratory, Agricultural Research Service, United States Department of Agriculture, c/o National Museum of Natural History, Smithsonian Institution, Washington, DC). These specimens are housed at the South African National Collection of Insects (A.R.C.), Pretoria, under the accession numbers AcP 9552 (Peru) and AcP 9553 (Brazil), and in the United States National Museum of Natural History, Washington, DC. Re‐examination of the original series and subsequent study of a much larger sample of specimens from each locality revealed a number of specific differences between the Peruvian and Brazilian specimens, including the width of the pronotum, the size and density of punctures and pubescence on the pronotum; and male genitalia, especially the width of the basal lobe on the left paramere and the shape of the middle process on the right paramere. These characters were found to consistently distinguish the two taxa, but the differences were subtle and without having had the reproductive incompatibility evidence and the information indicating large sequence divergences, these differences would have been considered interpopulation‐level morphological variation. Hybrid nymphs were used for genetic analyses before they developed to the adult stage so no morphological analysis of hybrids was possible.

Three of the 13 morphological features that were measured were significantly different between the two populations for male specimens (Table 1) and four were significantly different for female specimens (Table 2). The width of Brazilian males was slightly greater than that of Peruvian males for both body width as well as the width at the pronotum, but the average difference was less than 0.1 mm and overlaps in the size ranges for each population did occur (Table 1). The fourth antennal segment of Brazilian males was also 0.1 mm longer than that of Peruvian males but again overlaps in the size ranges of the two populations occurred (Table 1). No significant differences in the length of the paramere or the hook on the paramere were detected (Table 1). Brazilian females were significantly larger in length, width, pronotum width and head width, but the ranges of all parameters overlapped with the exception of width, and the ranges of this parameter were as close as 0.01 mm (Table 2). The lengths of the different body parts are therefore unlikely to be an accurate and consistent method to differentiate between the two taxa. Other morphological features, such as pubescence and puncturing of the pronotum as well as the shape of the male genetalia (see above), will need to be included in combination with body size to distinguish between the two populations. Differences between the measurements of the type specimen and those taken in this study can be attributed to the better accuracy with which the parameters were measured due to the more sophisticated microscopes and measuring programmes that are now available.

**Table 1:** Morphological measurements of male *Eccritotarsus catarinensis* from two source populations, as well as the measurements of the type specimen (Carvalho 1948)

Parameter	Carvalho (1948), mm	Brazil (±SE), mm	Peru (±SE), mm	Brazil range, mm	Peru range, mm	*t* Value	*P* value
Body length	3	2.87 (±0.063)	2.72 (±0.079)	2.68–3.36	2.52–3.39	−1.5	0.157
Body width	0.7	0.72 (±0.011)	0.63 (±0.009)	0.64–0.75	0.58–0.66	−6.3	**<0.001**
Pronotum length	0.7	0.72 (±0.007)	0.67 (±0.008)	0.64–0.84	0.61–0.74	−1.9	0.067
Pronotum width	1.3	0.81 (±0.011)	0.73 (±0.016)	0.76–0.84	0.69–0.77	−5.0	**<0.001**
Head length	0.2	0.23 (±0.006)	0.23 (±0.008)	0.20–0.28	0.20–0.27	0.1	0.978
Head width	0.5	0.58 (±0.005)	0.56 (±0.010)	0.55–0.68	0.52–0.69	−1.1	0.296
Head vertex	0.31	0.30 (±0.011)	0.31 (±0.011)	0.28–0.34	0.29–0.37	1.8	0.084
Antennae Segment 1	0.3	0.30 (±0.016)	0.29 (±0.015)	0.29–0.33	0.25–0.37	−1.2	0.265
Antennae Segment 2	0.5	0.55 (±0.015)	0.52 (±0.014)	0.47–0.59	0.46–0.58	−1.2	0.135
Antennae Segment 3	0.7	0.71 (±0.022)	0.69 (±0.014)	0.60–0.79	0.62–0.79	−0.9	0.375
Antennae Segment 4	0.6	0.64 (±0.012)	0.54 (±0.008)	0.57–0.70	0.50–0.65	−3.6	**0.002**
Length of left paramere	Not measured	0.22 (±0.041)	0.21 (±0.055)	0.20–0.24	0.18–0.22	1.5	0.151
Width of paramere hook	Not measured	0.04 (±0.016)	0.04 (±0.014)	0.03–0.05	0.03–0.05	−0.5	0.616

*P* values in bold indicate significant differences.

**Table 2:** Morphological measurements of female *Eccritotarsus catarinensis* from two source populations

Parameter	Brazil (±SE), mm	Peru (±SE), mm	Brazil range, mm	Peru range, mm	*t* Value	*P* value
Body length	2.95 (±0.031)	2.83 (±0.026)	2.79–3.17	2.73–2.92	−3.6	**0.007**
Body width	0.73 (±0.009)	0.66 (±0.010)	0.70–0.79	0.61–0.69	−5.6	**<0.001**
Pronotum length	0.44 (±0.023)	0.54 (±0.010)	0.32–0.50	0.38–0.48	−1.6	0.128
Pronotum width	0.83 (±0.010)	0.42 (±0.009)	0.79–0.88	0.73–0.83	−3.2	**0.004**
Head length	0.26 (±0.006)	0.26 (±0.010)	0.23–0.29	0.22–0.29	0.2	0.861
Head width	0.57 (±0.006)	0.54 (±0.005)	0.54–0.59	0.51–0.56	−3.1	**0.006**
Head vertex	0.30 (±0.008)	0.30 (±0.009)	0.27–0.36	0.26–0.33	−0.1	0.987
Antennae Segment 1	0.26 (±0.008)	0.3 (±0.005)	0.24–0.29	0.22–0.27	−2	0.059
Antennae Segment 2	0.45 (±0.005)	0.24 (±0.011)	0.44–0.55	0.41–0.53	−1	0.313
Antennae Segment 3	0.67 (±0.010)	0.48 (±0.010)	0.62–0.71	0.61–0.71	−0.2	0.871
Antennae Segment 4	0.60 (±0.009)	0.66 (±0.018)	0.46–0.70	0.50–0.66	−1.8	0.091

*P* values in bold indicate significant differences.

### No‐choice interbreeding experiment

Fourteen of the 15 replicates (93%) of Peru♀ × Peru♂ crosses produced offspring and 8 of 10 (80%) of the Brazil♀ × Brazil♂ crosses produced offspring. Only four of the 24 replicates (17%) of Brazil♀ × Peru♂ crosses produced any offspring and no offspring were produced by Peru♀ × Brazil♂ crosses.

**Figure 1:**
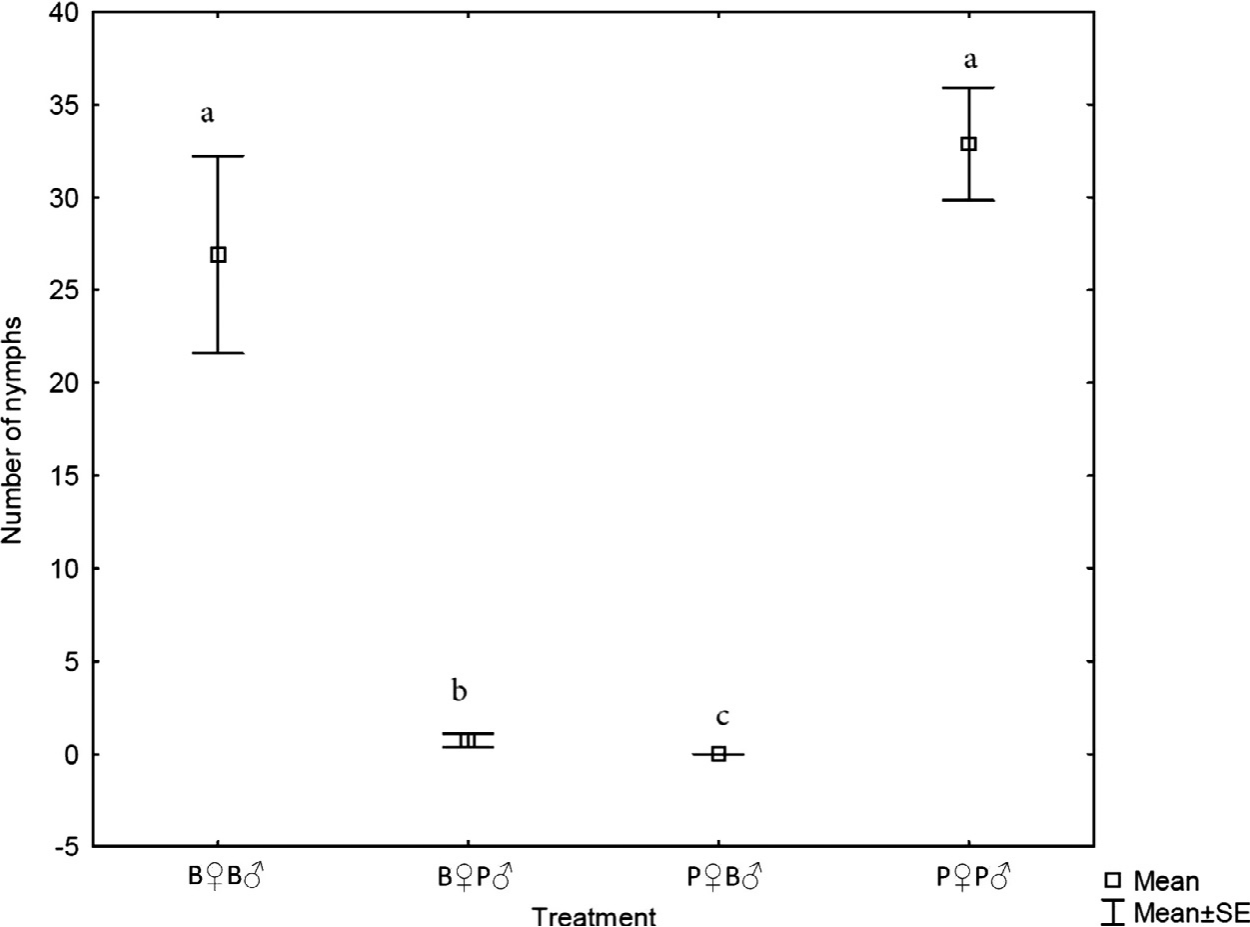
The median number of nymphs produced by breeding pairs for each interbreeding treatment under no‐choice conditions. Error bars represent the 25–75% range and lower case letters represent significant differences according to a Multiple Comparison of Mean Ranks test (*P* < 0.05) with the same letter representing no significant difference and different letters indicating where significant difference occurred. B indicates Brazilian population, P indicates the Peruvian population.

Crosses of Peru♀ × Peru♂ produced an average of 32.9 nymphs (SE ± 3.06) and crosses of Brazil♀ × Brazil♂ produced an average of 26.9 nymphs (SE ± 5.33). An average of only 0.67 nymphs (SE ± 0.34) was produced by Brazil♀ × Peru♂ crosses and no offspring resulted from the reciprocal cross of Peru♀ × Brazil♂ (Fig. 1). There was an overall significant difference between all treatments (*H* = 54.05; *P* < 0.00001), where the within‐population treatments (Brazil♀ × Brazil♂; Peru♀ × Peru♂) produced significantly more offspring than both interpopulation treatments (Brazil♀ × Peru♂; Peru♀ × Brazil♂). Multiple comparisons of means revealed no significant difference between the two within‐population treatments.

**Figure 2:**
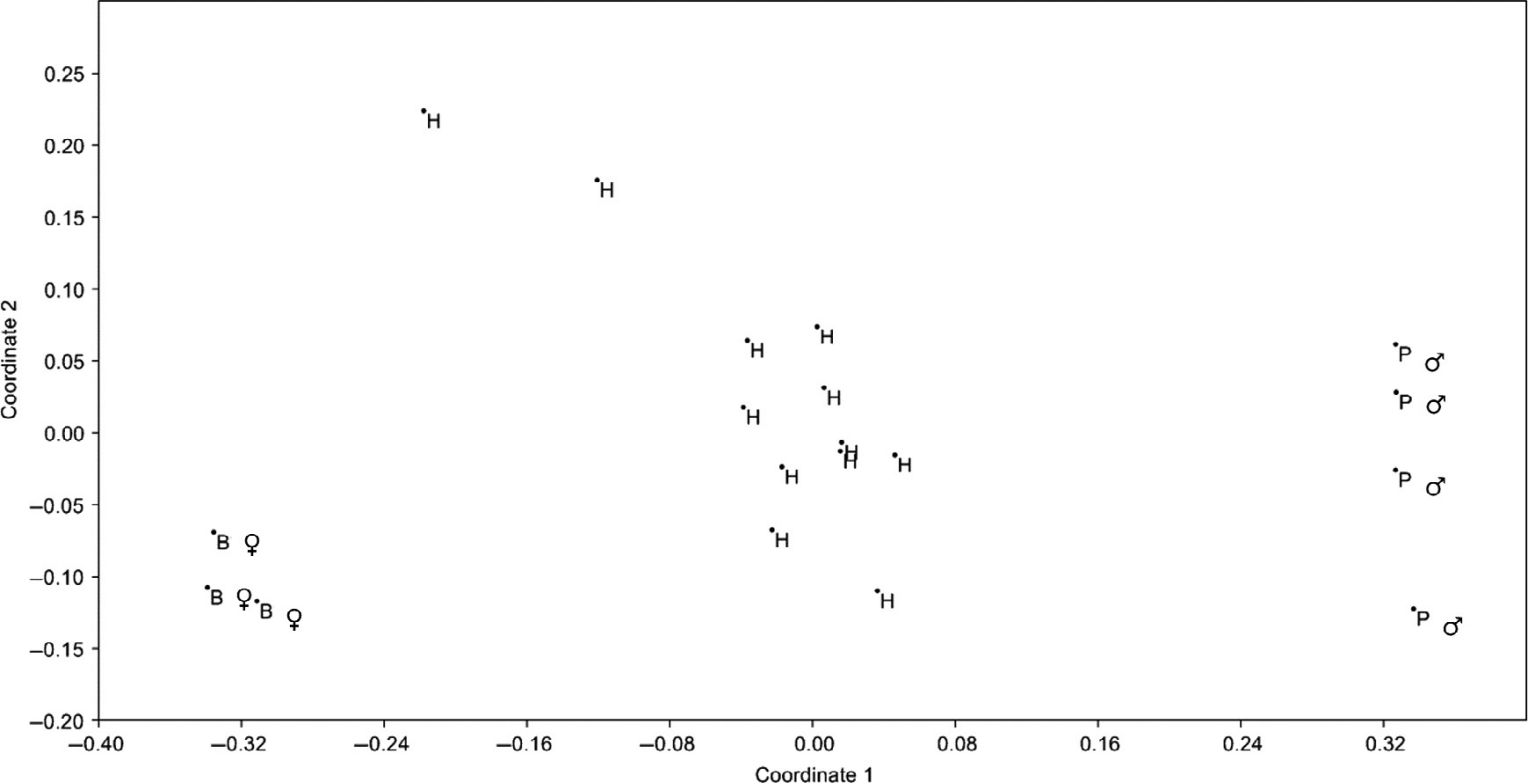
Non‐metric Multi‐Dimensional Scaling (nMDS) scatterplot using the ISSR data for hybrid offspring and the parents of hybrid offspring. Hybrids share an identical COI profile to their mothers and are intermediate to their parents in the MDS plot. B♀ indicates Brazilian mothers, P♂ the Peruvian fathers and H the hybrid offspring.

A total of 16 hybrid individuals were produced, 12 of which were analyzed using genetic techniques to confirm that they were true hybrids (see Genetic analysis for genetics methods). All hybrid individuals from the Brazil♀ × Peru♂ crosses shared the identical Brazilian COI sequence from Taylor et al. (2011) and formed a third group intermediate to the Brazilian and Peruvian parents in the nMDS from the ISSR data (Fig. 2).

### Choice interbreeding experiment

**Figure 3:**
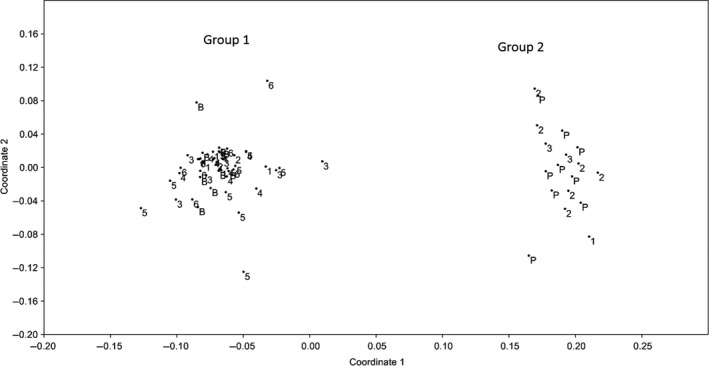
Non‐metric Multi‐Dimensional Scaling (nMDS) scatterplot using the ISSR data from the choice interbreeding experiment. ‘P’ and ‘B’ indicate individuals from the Brazilian and Peruvian control populations. Individuals from the six mixed populations are labeled 1–6. For the COI region, all individuals in Group 1 were Brazilian, all individuals in Group 2 were Peruvian.

Two clearly defined groups were formed in the nMDS scatterplot from the ISSR data with all the Brazilian controls falling into Group 1 and all the Peruvian controls into Group 2 (Fig. 3). All the individuals that formed Group 1 had identical COI sequences to the Brazilian COI sequences from Taylor et al. (2011) and all those in Group 2 had identical sequences to the Peruvian sequences from the same study (Genbank accession numbers: Brazilian KU530108; Peruvian KU530109). No individuals were intermediate between the two groups and all individuals within a group had identical COI sequences (Fig. 3). This indicates that no hybrids were sampled in this experiment. When hybrid individuals from the no‐choice experiment were included in this analysis, they formed an intermediate group to the Brazilian and Peruvian groups in the nMDS plot similar to the hybrid group in Figure 2.

Of the 50 individuals sampled from mixed populations, only nine grouped with the Peruvian samples (Group 2) and 41 grouped with the Brazilian samples (Group 1) (Fig. 3). Peruvian individuals were sampled in three of the six populations (one in Pop. 1, six in Pop. 2 and two in Pop. 3) whereas all six populations had Brazilian individuals (Fig. 3).

### Screening for *Wolbachia*

The *Wolbachia* control produced clear bands of about 600 bp as was expected for insects infected with the *Wolbachia* bacteria. No bands were obtained from any *E. catarinensis* individuals of either the Brazilian or Peruvian population, suggesting that neither of the populations was infected by *Wolbachia*.

## Discussion

Cryptic species can be recognized and delimited according to the biological species concept using various methods and types of data, but this study is one of the few that has evaluated levels of infertility and reproductive incompatibility between cryptic insect taxa using a combination of molecular information and crossbreeding experiments. The two species within *E. catarinensis* are reproductively isolated, but are so similar morphologically that they were previously considered representatives of a single species. As a consequence of our results, their morphological characteristics have been re‐appraised and a formal taxonomic description is being prepared to separate the cryptic species from Peru as a new species of *Eccritotarsus*. The new species can still be considered as cryptic because the subtle morphological differences that have been identified would previously have been considered as indicative of intraspecific variation.

One of the few other confirmed cryptic species is the sweet potato whitefly for which as many as 24 cryptic species were identified using genetic techniques (Dinsdale et al. 2010), many of which have been tested using crossbreeding experiments and confirmed as reproductively isolated species (Xu et al. 2010; Liu et al. 2012). There are many cases where possible cryptic species have been identified although DNA‐barcoding (Wilcox et al. 1997; Gómez et al. 2002; Hebert et al. 2004a,b; Miura et al. 2005; Ward et al. 2008; Saitoh et al. 2015) but because threshold percentage sequence divergence between species can differ dramatically between taxa, even within a single family, often overlapping with mean intraspecific divergence (no ‘barcoding gap’; Meyer and Paulay 2005; Cognato 2006; Park et al. 2011), these cannot be confidently confirmed as true cryptic species without verifying the levels of reproductive incompatibility between them. Other problems associated with DNA‐barcoding are the presence of mitochondrial DNA sequences in the nuclear genome (numts) and heteroplasmy (more than one mitochondrial genome; Song et al. 2008). The use of alternative data sources, such as ISSR, morphology and mating experiments in this study, can act as checks on these possible sources of error (Karanovic et al. 2016; Dejaco et al. In press), as can more laborious methodological controls, although such practices compromise barcoding's claim to be a fast and efficient method of species discovery and delimitation (Song et al. 2008).

Hybrid *E. catarinensis* nymphs could only be produced by Brazilian females under no‐choice conditions and the extremely low number of offspring produced suggests that it is possible that no hybrid lineages would persist under field conditions. The fact that no hybrids were sampled in the choice experiment may be due to the fact that hybrids are extremely rare and were therefore not sampled, or it is possible that when mates from the same species were available, no interbreeding between the two species occurred. No‐choice laboratory‐based experiments have consistently produced hybrids between well‐established species when interbreeding will not occur in wild populations (Liu et al. 2012), so the hybrid nymphs produced in this study are very likely to be artefacts of the no‐choice laboratory conditions under which the experiments were conducted. It is also possible that the small number of hybrid offspring produced in this study were sterile hybrids and could therefore not persist or increase in abundance over time (Coyne and Orr 1989). Hybrid sterility could not be tested in this study due to the low number of hybrids that were produced and because hybrids were killed to confirm their status using genetic methods. Hybrid sterility is believed to play an important role in the evolution of new species from allopatric populations (Orr 1995) and may therefore have contributed to divergence in the *E. catarinensis* system. Similarly, the lack of any hybrids produced by Peruvian females under both choice and no‐choice conditions may be due to complete incompatibility between Peruvian females and Brazilian males, or it is possible that hybrids are so rare that none were sampled in either experiment.

Little or no gene flow between *E. catarinensis* populations in Peru and Brazil would be expected due to the large geographic distance that separates the two populations. *Eccritotarsus catarinensis* has been recorded in the states of Santa Catarina (Carvalho 1948) and Rio de Janeiro (Hill et al. 1999) in southern Brazil and in Loreto Province on the Yurapa River in northern Peru (Cordo 1999). No populations of *E. catarinensis* have been recorded between southern Brazil and northern Peru but it is possible that populations do exist. The lack of gene flow between the two populations may have resulted in independent mutations that led to reproductive barriers with limited morphological changes. Alternatively, the genetic bottleneck that the Brazilian population experienced after importation into South Africa, and subsequent population increase under relaxed selective conditions, may have resulted in the formation of a new species – a founder‐flush effect (Carson 1971; Powell 1978). After importation into quarantine in South Africa, all but one gravid female died (Taylor et al. 2011). In addition, there has been no opportunity for subsequent gene flow with the source population, there is no evidence of any bottlenecks in the source population, and the founding population was introduced into a habitat different from the source. These conditions are thought to be required for founder effect or founder‐flush speciation (Giddings and Templeton 1983; Whitlock 1997; Fauergue et al. 2012) and have been present in supporting examples (Regan et al. 2003; Matute 2013; Oh et al. 2013). In addition, the population had human assistance in surviving the bottleneck, overcoming the large extinction probability in extremely small populations. It is possible that a genetic reorganization in that gravid female and her descendants render mating incompatible with the Peruvian, as well as Brazilian individuals in the source population. This hypothesis predicts reproductive isolation between the South African population derived from Brazil and that population in the native range. This could be tested by collecting *E. catarinensis* from the original collection site of the Brazilian population in Florianopolis, Santa Catarina, and conducting interbreeding experiments between that population and individuals from the two species in South Africa. The founder effect and founder‐flush models of speciation have received criticism (Barton and Charlesworth 1984; Coyne and Orr 2004) as well as support (Slatkin 1996; Templeton 2008; Wessel et al. 2013; Pierce et al. 2016), so further study to determine whether these theories have played a role in the evolution of *Eccritotarsus* is warranted.

Few biological control agents for the control of invasive alien plants are screened using genetic techniques prior to release but there are some records of cryptic species being discovered in biological control agent populations or in populations of insects being considered as potential agents (Tošveski et al. 2013). Morphologically identical populations of the flea beetle, *Psylliodes chalcomerus* (Illiger) [Coleoptera: Chrysomelidae], a potential biological control agent for yellow starthistle, *Centaurea solstitialis* L. [Asteraceae], having a variety of different host plants in the indigenous range, were all regarded as a single species but were divided into three possible cryptic species using genetic analyses (Gaskin et al. 2011). Tošveski et al. (2013) also identified cryptic species within the indigenous range of the *Mecinus janthinus* Germar [Coleoptera: Curculionidae] species complex, a weevil introduced into North America as a biological control agent for two species of toadflax (*Linaria* spp. [Scrophulariaceae]). Whether the cryptic species of *M. janthinus* are present in the introduced populations in North America has not been confirmed (Tošveski et al. 2013). A possible cryptic species of the flea beetle, *Aphthona lacertosa* Rosenheim [Coleoptera: Chrysomelidae], a biological control agent for leafy spurge, *Euphorbia esula* L. [Euphorbiaceae], was identified using genetic techniques after its release in North America (Roehrdanz et al. 2011). The leafy spurge flea beetle is the only example of a cryptic species of weed biological control agent that was discovered after release besides *E. catarinensis*.

The existence of cryptic species in biological control agent populations could result in unpredicted nontarget effects if consignments of the agents that are exported from the native range are assumed to have identical host ranges. Paynter et al. (2008) discovered that two populations of the gorse pod moth, *Cydia succedana* (Denis and Schiffermüller) [Lepidoptera: Olethreutidae], a biological control agent for the invasive alien plant gorse, *Ulex europaeus* L. [Fabaceae], in New Zealand, differed in their host ranges. The two populations were sourced from geographically isolated populations in the native range of the moth but the low sequence divergence indicated that the two populations were not cryptic species (Paynter et al. 2008). With a higher sequence divergence between the two *Eccritotarsus* species, there is a higher likelihood that the host ranges of the species could differ (Taylor et al. 2011) and host specificity data indicate that there are subtle differences in the host ranges of the two species (I.D. Paterson, unpublished data). Fortunately, both populations were subjected to host specificity testing before being released in South Africa despite the fact that host specificity testing of the Peruvian consignment was technically not required because the species had already been cleared for release (I.D. Paterson, unpublished data). Both species are safe for release in South Africa and no nontarget effects are predicted because, although there are subtle differences in the host ranges, these differences are restricted to close relatives of the target plant that are not indigenous or grown commercially in South Africa. Although in this case the two consignments of the agent were both subjected to host specificity testing, this is not necessarily common practice in all biological control programmes. The fact that the two consignments of the agent appear to have slightly different host ranges indicates that the practice of importing multiple consignments of biological control agents for the purpose of increasing genetic diversity of agent populations should be conducted with extreme caution (Paynter et al. 2008). All consignments, especially those collected from allopatric populations, should be treated as distinct entities with potentially different host ranges until proven otherwise. Different populations of the same species may have different host ranges due to local adaptations (Karban 1989, 1992; Hanks and Denno 1994) and in extreme cases, each population may be a cryptic species. All consignments should also be screened for the existence of cryptic species using basic genetic analyses such as sequencing the COI region of a subset of consignments.

Because both species occupy a similar ecological niche, there is the possibility of competition between the two species if both are released as biological control agents. From the choice interbreeding experiment in this study, it was clear that the Brazilian species had become more abundant than the Peruvian species because the majority of the samples taken were Brazilian. This suggests that the Brazilian species was a better competitor under those conditions. Temperature is likely to be an important factor in determining which of the two species is a stronger competitor because the Peruvian species was collected from a much warmer region than the Brazilian. The Peruvian species should therefore be released at sites in warm, tropical regions and the Brazilian species in cooler more temperate regions. Further study of the thermal physiologies of both species are, however, required.

The mechanisms that cause reproductive isolation between the two species have not been identified. The genitalia, and more specifically the parameres, of *Eccritotarsus* are used as characters to define the various species in the genus (Carvalho 1948) and there are only relatively small differences in these features between the two species but the small differences may be sufficient to prevent sperm transfer. The bacterium *Wolbachia* has been ruled out as a cause of the reproductive isolation in this study because it is not present in either species. Preliminary studies suggest that premating behavioral barriers may be involved and studies are now underway to determine rates of copulation events between the two species under choice and no‐choice conditions, whether sperm transfer is successful for between‐species matings, and whether pheromones may play a role in reproductive isolation. Speciation has occurred but the speciation has resulted in very limited morphological change. The changes that have resulted in reproductive isolation of the two species may therefore be present in some other characteristic that could be physical or behavioral (Bickford et al. 2007).

## Conflict of Interest

None declared.

## References

[ece32297-bib-0001] Abad‐Franch, F. , M. G. Pavan , N. Jaramillo‐O , F. S. Palomeque , C. Dale , D. Chaverra , et al. 2013 *Rhodnius barretti*, a new species of Triatominae (Hemiptera: Reduviidae) from western Amazonia. Mem. Inst. Oswaldo Cruz 108(Suppl. 1):92–99. 24473808 10.1590/0074-0276130434 PMC4109185

[ece32297-bib-0002] Abbot, P. 2001 Individual and population variation in invertebrates revealed by Inter‐simple Sequence Repeats (ISSRs). J. Insect Sci. 1:1–3. 15455068 PMC355892

[ece32297-bib-0003] Arrigo, N. , J. W. Tuszynski , D. Ehrich , T. Gerdes , and N. Alvarez . 2009 Evaluating the impact of scoring parameters on the structure of intra‐specific genetic variation using RawGeno, an R package for automating AFLP scoring. BMC Bioinformatics 23:34–56. 10.1186/1471-2105-10-33 PMC2656475 19171029

[ece32297-bib-0004] Barton, N. , and B. Charlesworth . 1984 Genetic revolutions, founder effects, and speciation. Annu. Rev. Ecol. Syst. 15:133–164.

[ece32297-bib-0005] Bickford, D. , D. J. Lohman , S. S. Navjot , P. K. L. Ng , R. Meier , R. Winker , et al. 2007 Cryptic species as a window on diversity and conservation. Trends Ecol. Evol. 22:148–155. 17129636 10.1016/j.tree.2006.11.004

[ece32297-bib-0006] Bordenstein, S. R. , F. P. O'Hara , and J. H. Werren . 2001 *Wolbachia*‐induced incompatibility precedes other hybid incompatibilities in *Nasonia* . Nature 409:707–710. 11217858 10.1038/35055543

[ece32297-bib-0007] Carson, H. L. 1971 Speciation and the founder principle. Stadler Genet. Symp. Univ. Missouri 3:51–70.

[ece32297-bib-0008] Carvalho, J. C. M. 1948 Mírideos neotropical, XXXI: Gênero “*Pseudobryocoris*” Distant e uma espécie nova de “Neella” Reuter (Hemiptera). An. Acad. Bras. Ciênc. 54:95–104.

[ece32297-bib-0009] Coetzee, J. A. , M. J. Byrne , and M. P. Hill . 2007a Predicting the distribution of *Eccritotarsus catarinensis*, a natural enemy released on water hyacinth in South Africa. Entomol. Exp. Appl. 125:237–247.

[ece32297-bib-0010] Coetzee, J. A. , M. J. Byrne , and M. P. Hill . 2007b Impacts of nutrients and herbivory by *Eccritotarsus catarinensis* on the biological control of water hyacinth, *Eichhornia crassipes* . Aquat. Bot. 86:179–186.

[ece32297-bib-0011] Coetzee, J. A. , M. P. Hill , M. P. Byrne , and A. Bownes . 2011 A review of the biological control programmes on *Eichhornia crassipes* (C. Mart.) Solms (Pontederiaceae), *Salvinia molesta* D.S. Mitch. (Salviniaceae), *Pistia stratiotes* L. (Araceae), *Myriophyllum aquaticum* (Vell.) Verdc. (Haloragaceae) and *Azolla filiculoides* Lam. (Azollaceae) in South Africa since 1999. Afr. Entomol. 19:451–468.

[ece32297-bib-0012] Cognato, A. I. 2006 Standard percent DNA sequence difference for insects does not predict species boundaries. J. Econ. Entomol. 99:1037–1045. 16937653 10.1603/0022-0493-99.4.1037

[ece32297-bib-0013] Colautti, R. I. , M. Manca , M. Vijanen , H. A. M. Ketelaars , H. Bürgi , H. J. Macisaac , et al. 2005 Invasion genetics of the Eurasian spiny waterflea: evidence for bottlenecks and gene flow using microsatellites. Mol. Ecol. 14:1869–1879. 15910312 10.1111/j.1365-294X.2005.02565.x

[ece32297-bib-0014] Cook, L. G. , R. D. Edwards , M. D. Crisp , and N. B. Hardy . 2010 Need morphology always be required for new species descriptions? Invertebr. Syst. 24:322–326.

[ece32297-bib-0015] Cordo, H. A. 1999 New agents for biological control of water hyacinth Pp. 68–74 *in* Hill M. P., Julien M. H. and Center T. D., eds. Proceedings of the first IOBC Global Working Group Meeting for the Biological and Integrated Control of Water Hyacinth, 16–19 November 1998, St Lucia Park Hotel, Harare, Zimbabwe. Plant Protection Research Institute, Pretoria.

[ece32297-bib-0016] Coyne, J. A. , and H. A. Orr . 1989 Patterns of speciation in *Drosophila* . Evolution 43:362–381. 10.1111/j.1558-5646.1989.tb04233.x 28568554

[ece32297-bib-0017] Coyne, J. A. , and H. A. Orr . 2004 Speciation. Sinauer Associates, Sunderland, MA 545 Pp.

[ece32297-bib-0018] Darlington, C. D. 1940 Taxonomic systems and genetic systems Pp. 137–160 *in* Huxley J., ed. The new systematics. Clarendon Press, Oxford, U.K.

[ece32297-bib-0019] Davis, M. A. , M. R. Douglas , M. L. Collyer , and M. E. Douglas . 2016 Deconstructing a species‐complex: geometric morphometric and molecular analyses define species in the Western Rattlesnake (*Crotalus viridis*). PLoS One 11:e0146166. 26816132 10.1371/journal.pone.0146166 PMC4731396

[ece32297-bib-0020] Dejaco, T. , M. Gassner , W. Arthofer , B. C. Schlick‐Steiner , and F. M. Steiner . In press. Taxonomist's nightmare … evolutionist's delight: an integrative approach resolves species limits in jumping bristletails despite widespread hybridization and parthenogenesis. Syst. Biol. 10.1093/sysbio/syw003 PMC5066060 26869489

[ece32297-bib-0021] Dinsdale, A. , L. Cook , C. Riginos , Y. M. Buckley , and P. J. de Barro . 2010 Refined global analysis of *Bemisia tabaci* (Gennadius) (Hemiptera: Sternorrhyncha: Aleyrodidae) mitochondrial COI to identify species level genetic boundaries. Ann. Entomol. Soc. Am. 103:196–208.

[ece32297-bib-0022] Fauergue, X. , E. Vercken , T. Malausa , and R. A. Hufbauer . 2012 The biology of small, introduced populations, with special reference to biological control. Evol. Appl. 5:424–443. 22949919 10.1111/j.1752-4571.2012.00272.x PMC3407862

[ece32297-bib-0023] Gaskin, J. F. , M. C. Bon , M. J. W. Cock , M. Cristofaro , A. De Biase , R. De Clerck‐Floate , et al. 2011 Applying molecular‐based approaches to classical biological control of weeds. Biol. Control 58:1–21.

[ece32297-bib-0024] Giddings, L. V. , and A. R. Templeton . 1983 Behavioral phylogenies and the direction of evolution. Science 220:372–378. 17831399 10.1126/science.220.4595.372

[ece32297-bib-0025] Gómez, A. , M. Serra , G. R. Carvalho , and D. H. Lunt . 2002 Speciation in ancient cryptic species complexes: evidence from the molecular phylogeny of *Brachionus plicatilis* (Rotifera). Evolution 56:1431–1444. 12206243 10.1111/j.0014-3820.2002.tb01455.x

[ece32297-bib-0026] Hammer, Ø. , and D. A. T. Harper . 2001 PAST: paleontological statistics software package for education and data analysis. Paleontol. Electronica 4:1–9 (Art 4).

[ece32297-bib-0027] Hanks, L. M. , and R. F. Denno . 1994 Local adaptation in the armored scale insect *Pseudaulacaspis pentagona* (Homoptera: Diaspididae). Ecology 75:2301–2310.

[ece32297-bib-0028] Hebert, P. D. N. , A. Cywinski , S. L. Ball , and J. R. de Waard . 2003 Biological identifications through DNA barcodes. Proc. R. Soc. Lond. B Biol. Sci. 270:313–321. 10.1098/rspb.2002.2218 PMC1691236 12614582

[ece32297-bib-0029] Hebert, P. D. N. , G. H. Penton , J. M. Burns , D. H. Janzen , and W. Hallwachs . 2004a Ten species in one: DNA barcoding reveals cryptic species in the Neotropical skipper butterfly *Astraptes fulgerator* . PNAS 101:14812–14817. 15465915 10.1073/pnas.0406166101 PMC522015

[ece32297-bib-0030] Hebert, P. D. , M. Y. Stoeckle , T. S. Zemlak , and C. M. Francis . 2004b Identification of birds through DNA barcodes. PLoS Biol. 2:E312. 15455034 10.1371/journal.pbio.0020312 PMC518999

[ece32297-bib-0031] Hill, M. P. , C. J. Cilliers , and S. Neser . 1999 Life history and laboratory host range of *Eccritotarsus catarinensis* (Carvalho) (Hemiptera: Miridae), a new natural enemy released on water hyacinth (*Eichhornia crassipes* (Mart.) Solms‐Laub.) (Pontederiaceae) in South Africa. Biol. Control 14:127–133.

[ece32297-bib-0032] Kaiser, L. , B. P. Le Ru , F. Kaoula , C. Paillusson , C. Capdevielle‐Dulac , J. O. Obonyo , et al. 2015 Ongoing ecological speciation in *Cotesia sesamiae*, a biological control agent of cereal stem borers. Evol. Appl. 8:807–820. 26366198 10.1111/eva.12260 PMC4561570

[ece32297-bib-0033] Karanovic, T. , M. Djurakic , and S. M. Eberhard . 2016 Cryptic species or inadequate taxonomy? Implementation of 2D geometric morphometrics based on integumental organs as landmarks for delimitation and description of copepod taxa. Syst. Biol. 65:304–327. 26608965 10.1093/sysbio/syv088

[ece32297-bib-0034] Karban, R. 1989 Fine‐scale adaptation of herbivorous thrips to individual host plants. Nature 340:60–61.

[ece32297-bib-0035] Karban, R. 1992 Plant variation: its effects on populations of herbivorous insects Pp. 195–215 *in* Fritz R. S. and Simms E. L., eds. Plant resistance to herbivores and pathogens. Ecology, evolution and genetics. The Univ. of Chicago Press, Chicago and London.

[ece32297-bib-0036] Lachmuth, S. , W. Durka , and F. M. Schurr . 2010 The making of a rapid plant invader: genetic diversity and differentiation in the native and invaded range of *Senecio inaequidens* . Mol. Ecol. 19:3952–3967. 20854275 10.1111/j.1365-294X.2010.04797.x

[ece32297-bib-0037] Liu, S. S. , J. Colvin , and P. J. De Barro . 2012 Species concepts as applied to the whitefly *Bemisia tabaci* systematics: how many species are there? J. Integr. Agric. 11:176–186.

[ece32297-bib-0038] Machtelinckx, T. , T. Van Leeuwen , B. Vanholme , B. Gehesquière , W. Dermauw , B. Vandekerkhove , et al. 2009 *Wolbachia* induces strong cytoplasmic incompatibility in the predatory bug *Macrolophus pygmaeus* . Insect Mol. Biol. 18:373–381. 19523069 10.1111/j.1365-2583.2009.00877.x

[ece32297-bib-0039] Matute, D. R. 2013 The role of founder effects on the evolution of reproductive isolation. J. Evol. Biol. 26:2299–2311. 24118666 10.1111/jeb.12246

[ece32297-bib-0040] Mayr, E. 1942 Systematics and the origin of species. Columbia Univ. Press, New York.

[ece32297-bib-0041] Mayr, E. 1963 Animal species and evolution. The Belknap Press of Harvard Univ. Press, Cambridge, U.K.

[ece32297-bib-0042] Meyer, C. P. , and G. Paulay . 2005 DNA barcoding: error rates based on comprehensive sampling. PLoS Biol. 3:e422. 16336051 10.1371/journal.pbio.0030422 PMC1287506

[ece32297-bib-0043] Miura, O. , A. M. Kuris , M. E. Torchin , R. F. Hechinger , E. J. Dunham , and S. Chiba . 2005 Molecular‐genetic analysis reveals cryptic species trematodes of intertidal gastropod, *Batillaria cumingi* (Crosse). Int. J. Parasitol. 35:793–801. 15925598 10.1016/j.ijpara.2005.02.014

[ece32297-bib-0044] Noor, M. A. F. 2002 Is the biological species concept showing its age? Trends Ecol. Evol. 17:153–154.

[ece32297-bib-0045] Oh, K. P. , G. L. Conte , and K. L. Shaw . 2013 Founder effects and the evolution of asymmetrical sexual isolation in a rapidly‐speciating clade. Curr. Zool. 59:230–238.

[ece32297-bib-0046] Orr, H. A. 1995 The population genetics of speciation: the evolution of hybrid incompatibilities. Genetics 139:1805–1813. 7789779 10.1093/genetics/139.4.1805 PMC1206504

[ece32297-bib-0047] Park, D. S. , R. Foottit , E. Maw , and P. D. N. Hebert . 2011 Barcoding bugs: DNA‐based identification of the true bugs (Insecta: Hemiptera: Heteroptera). PLoS One 6:e18749. 21526211 10.1371/journal.pone.0018749 PMC3078146

[ece32297-bib-0048] Paynter, Q. , A. H. Gourlay , P. T. Oboyski , S. V. Fowler , R. L. Hill , T. M. Withers , et al. 2008 Why did specificity testing fail to predict the field host‐range of the gorse pod moth in New Zealand? Biol. Control 46:453–462.

[ece32297-bib-0049] Pierce, A. A. , M. P. Zalucki , M. Bangura , M. Udawatta , M. R. Kronforst , S. Altizer , et al. 2016 Serial founder effects and genetic differentiation during worldwide range expansion of monarch butterflies. Proc. R. Soc. Lond. B Biol. Sci. 281:20142230. 10.1098/rspb.2014.2230 PMC4241002 25377462

[ece32297-bib-0050] Powell, J. R. 1978 The founder‐flush speciation theory: an experimental approach. Evolution 32:465–474. 10.1111/j.1558-5646.1978.tb04589.x 28567948

[ece32297-bib-0051] Regan, J. L. , L. M. Meffert , and E. H. Bryant . 2003 A direct experimental test of founder‐flush effects on the evolutionary potential for assortative mating. J. Evol. Biol. 16:302–312. 14635869 10.1046/j.1420-9101.2003.00521.x

[ece32297-bib-0052] Rissler, L. J. , and J. J. Apodaca . 2007 Adding more ecology into species delimitation: ecological niche models and phylogeography help define cryptic species in the Black Salamander (*Aneides flavipunctatus*). Syst. Biol. 56:924–942. 18066928 10.1080/10635150701703063

[ece32297-bib-0053] Roehrdanz, R. , R. Bourchier , A. Cortilet , D. Olson , and S. Sears . 2011 Phylogeny and genetic diversity of flea beetles (*Aphthona sp*.) introduced to North America as biological control agents for leafy spurge. Ann. Entomol. Soc. Am. 104:966–975.

[ece32297-bib-0054] Ross, K. G. , and D. D. Shoemaker . 2005 Species delimitation in native South American fire ants. Mol. Ecol. 14:3419–3438. 16156813 10.1111/j.1365-294X.2005.02661.x

[ece32297-bib-0055] Saitoh, T. , N. Sugita , S. Someya , Y. Iwami , S. Kobayashi , H. Kamigaichi , et al. 2015 DNA barcoding reveals 24 distinct lineages as cryptic bird species candidates in and around the Japanese Archipelago. Mol. Ecol. 15:177–186. 10.1111/1755-0998.12282 24835119

[ece32297-bib-0056] Schlick‐Steiner, B. C. , B. Seifert , C. Stauffer , E. Christian , R. H. Crozier , and F. M. Steiner . 2007 Without morphology, cryptic species stay in taxonomic crypsis following discovery. Trends Ecol. Evol. 22:391–392. 17573150 10.1016/j.tree.2007.05.004

[ece32297-bib-0057] Simões, P. M. , G. Mialdea , D. Reiss , M.‐F. Sagot , and S. Charlat . 2011 Wolbachia detection: an assessment of standard PCR protocols. Mol. Ecol. Resour. 11:567–572. 21481216 10.1111/j.1755-0998.2010.02955.x

[ece32297-bib-0058] Slatkin, M. 1996 In defense of founder‐flush theories of speciation. Am. Nat. 147:493–505.

[ece32297-bib-0059] Song, H. , J. E. Buhay , M. F. Whiting , and K. A. Crandall . 2008 Many species in one: DNA barcoding overestimates the number of species when nuclear mitochondrial pseudogenes are coamplified. Proc. Natl Acad. Sci. USA 105:13486–13491. 18757756 10.1073/pnas.0803076105 PMC2527351

[ece32297-bib-0060] Souza, N. A. , C. A. Andrade‐Coelho , F. M. Vigoder , R. D. Ward , and A. A. Peixoto . 2008 Reproductive isolation between sympatric and allopatric Brazilian populations of *Lutzomyia longipalpis s.l*. (Diptera: Psychodidae). Mem. Inst. Oswaldo Cruz 103:216–219. 18425278 10.1590/s0074-02762008000200017

[ece32297-bib-0061] Taylor, S. J. , D. A. Downie , and I. D. Paterson . 2011 Genetic diversity of introduced populations of the water hyacinth biological control agent *Eccritotarsus catarinensis* (Hemiptera: Miridae). Biol. Control 58:330–336.

[ece32297-bib-0062] Templeton, A. R. 2008 The reality and importance of founder speciation in evolution. BioEssays 30:470–479. 18404703 10.1002/bies.20745

[ece32297-bib-0063] Tošveski, I. , J. Jovic , O. Krstic , and A. Gassmann . 2013 PCR‐RFLP‐based method for reliable discrimination of cryptic species within *Mecinus janthinus* species complex (Mecinini, Curculionidae) introduced in North America for biological control of invasive toadflaxes. Biocontrol 58:563–573.

[ece32297-bib-0064] Ward, R. D. , B. H. Holmes , and G. K. Yearsley . 2008 DNA barcoding reveals a likely second species of Asian sea bass (barramundi) (*Lates calcarifer*). J. Fish Biol. 72:458–463.

[ece32297-bib-0065] Wessel, A. , H. Hoch , M. Asche , T. von Rintelen , B. Stelbrink , V. Heck , et al. 2013 Founder effects initiated rapid species radiation in Hawaiian cave planthoppers. Proc. Natl Acad. Sci. USA 110:9391–9396. 23696661 10.1073/pnas.1301657110 PMC3677470

[ece32297-bib-0066] Whitlock, M. C. 1997 Founder effects and peak shifts without genetic drift: adaptive peak shifts occur easily when environments fluctuate slightly. Evolution 51:1044–1048. 10.1111/j.1558-5646.1997.tb03951.x 28565491

[ece32297-bib-0067] Wilcox, T. P. , L. Hugg , J. A. Zeh , and D. W. Zeh . 1997 Mitochondrial DNA sequencing reveals extreme genetic differentiation in a cryptic species complex of Neotropical pseudoscorpions. Mol. Phylogenet. Evol. 7:208–216. 9126563 10.1006/mpev.1996.0388

[ece32297-bib-0068] Winston, R. L. , M. Schwarzländer , H. L. Hinz , M. D. Day , M. J. Cock , and M. H. Julien . 2014 Biological control of weeds: a world catalogue of agents and their target weeds. 5th ed USDA Forest Service, Forest Health Technology Enterprise Team, Morgantown, West Virginia FHTET‐2014‐04. 838 Pp.

[ece32297-bib-0069] Xu, J. , P. J. de Barro , and S. S. Liu . 2010 Reproductive incompatibility among genetic groups of *Bemisia tabaci* supports the proposition that the whitefly is a cryptic species complex. Bull. Entomol. Res. 100:359–366. 20178675 10.1017/S0007485310000015

[ece32297-bib-0070] Zhou, W. , F. Rousset , and S. O'Neil . 1998 Phylogeny and PCR‐based classification of Wolbachia strains using wsp gene sequences. Proc. R. Soc. Lond. B Biol. Sci. 265:509–515. 10.1098/rspb.1998.0324 PMC1688917 9569669

